# Quantification of Cell Edge Velocities and Traction Forces Reveals Distinct Motility Modules during Cell Spreading

**DOI:** 10.1371/journal.pone.0003735

**Published:** 2008-11-17

**Authors:** Benjamin J. Dubin-Thaler, Jake M. Hofman, Yunfei Cai, Harry Xenias, Ingrid Spielman, Anna V. Shneidman, Lawrence A. David, Hans-Günther Döbereiner, Chris H. Wiggins, Michael P. Sheetz

**Affiliations:** 1 Department of Biological Sciences, Columbia University, New York, New York, United States of America; 2 Cell Motion Laboratories, Inc., New York, New York, United States of America; 3 Department of Physics, Columbia University, New York, New York, United States of America; 4 Department of Applied Physics and Applied Math, Columbia University, New York, New York, United States of America; 5 Department of Biomedical Engineering, Columbia University, New York, New York, United States of America; 6 Universität Bremen, Institut für Biophysik, Bremen, Germany; University of Birmingham, United Kingdom

## Abstract

Actin-based cell motility and force generation are central to immune response, tissue development, and cancer metastasis, and understanding actin cytoskeleton regulation is a major goal of cell biologists. Cell spreading is a commonly used model system for motility experiments – spreading fibroblasts exhibit stereotypic, spatially-isotropic edge dynamics during a reproducible sequence of functional phases: 1) During early spreading, cells form initial contacts with the surface. 2) The middle spreading phase exhibits rapidly increasing attachment area. 3) Late spreading is characterized by periodic contractions and stable adhesions formation. While differences in cytoskeletal regulation between phases are known, a global analysis of the spatial and temporal coordination of motility and force generation is missing. Implementing improved algorithms for analyzing edge dynamics over the entire cell periphery, we observed that a single domain of homogeneous cytoskeletal dynamics dominated each of the three phases of spreading. These domains exhibited a unique combination of biophysical and biochemical parameters – a *motility module*. Biophysical characterization of the motility modules revealed that the early phase was dominated by periodic, rapid membrane blebbing; the middle phase exhibited continuous protrusion with very low traction force generation; and the late phase was characterized by global periodic contractions and high force generation. Biochemically, each motility module exhibited a different distribution of the actin-related protein VASP, while inhibition of actin polymerization revealed different dependencies on barbed-end polymerization. In addition, our whole-cell analysis revealed that many cells exhibited heterogeneous combinations of motility modules in neighboring regions of the cell edge. Together, these observations support a model of motility in which regions of the cell edge exhibit one of a limited number of motility modules that, together, determine the overall motility function. Our data and algorithms are publicly available to encourage further exploration.

## Introduction

Acto-myosin-based cell motility plays a central role in diverse cellular processes such as immune response [1], [2], wound healing [3], development [4]–[6], and cancer metastasis [7], [8]. While cytoskeletal motility depends on cellular context, the essential cytoskeletal proteins are conserved across eukaryotes [9]. This similarity may explain why similar motility phenotypes such as blebbing (membrane protrusion following dissociation with the cytoskeleton), ruffling, filopodia (long, thin actin bundles), and lamellipodia (broad, thin membrane extensions) are observed across a broad range of cells such as mouse fibroblasts, endothelial cells, T-cells, neuronal cells, mammalian and amphibian epithelial cells, and drosophila wing-disk cells [10]–[14]. We conjectured that these similarities in phenotype arise from a limited number of stable, biochemical and biophysical states of the cytoskeleton, or *motility modules*. Coordinated up and down-regulation of particular motility modules could give rise to functional states of the cell, and, since there are a limited number of motility modules, would also explain the observation that there is a limited number of steady-state cell morphologies [15]. Even if there exist only a small number of motility modules, by combining different motility modules the cell could achieve a larger number of functions, e.g., combining protrusive motility modules on one side of the cell with retraction on the other gives rise to migration. This concept can simplify biophysical modeling of cell motility by viewing the process as a sum of currently active motility modules.

Fibroblast motility is experimentally greatly simplified during cell spreading assays. A major difficulty in understanding the biophysical and biochemical bases of fibroblast motility stems from the wide variety of motility these cells can display at a given time. During the process of migration alone, fibroblasts exhibit lamellipodial protrusion and retraction, filopodial protrusion and retraction, bleb protrusion and retraction, trailing edge retraction, ruffling, and quiescence. We propose that each of these organizations of the cytoskeleton represents a single motility module and that the precise spatial and temporal organization of these various motility modules will lead to a particular cell function, be it chemotactic migration, phagocytosis, or post-mitotic spreading. However, measuring biophysical and biochemical parameters in the heterogeneous situation of cell migration is a difficult task.

Happily, it has long been understood that “the spreading of cultured cells on the substratum may be regarded as a prototype of a major group of morphogenetic processes by which cells acquire non-spherical shapes and become attached to extracellular matrices,” [16] and that cell spreading is a simple, physiologically-relevant method for isolating cytoskeletal behavior from the myriad of other cellular processes. Cell spread area as a function of time is well described by a sigmoid curve [17], and the spread area is a widely used statistic for establishing the role a particular molecule or disease state plays in cytoskeletal regulation [18]–[22]. Detailed light and electron microscope analyses have revealed that each temporal domain of the sigmoid corresponds to a distinct phase of spreading [23], [24], where the early phase (P0) consists of initial contact formation, the middle phase (P1) consists of fast contact area increase, and the final phase (P2) consists of slower protrusion and eventual polarization. Our previous quantitative analyses showed that limited sections of the cell periphery undergo an abrupt change in edge kinematics between the middle and late phases of cell spreading [25]. In addition, we previously found that membrane movement was highly uniform over the entire periphery of *isotropically* spreading cells [26] in the middle, fast phase of spreading. Thus, cell spreading provides an experimental system in which the normally heterogeneous cytoskeleton can be modeled by a reproducible temporal progression of functional phases, that are, at least in middle spreading, spatially homogenous. We propose that each phase of spreading represents a distinct cell-function and will exhibit a specific combination of motility modules.

To test this hypothesis, accurate tools for identifying motility modules over the entire cell periphery during spreading are required. One such tool, used to display and quantify edge dynamics during cell spreading, is the *edge velocity map* [26], a two-dimensional analog of the kymograph. The velocity map is a plot of normal velocity as a function of arc-length and time (normal velocity is defined as the speed of edge movement in the direction normal to the edge). Velocity maps have been used to evaluate the kinematics of cell motility over a variety of cell types and conditions [10], [12], [14], [25]–[28]. Interestingly, the dynamics of the filopodial-rich neuronal growth cone [29] were recently found to exhibit an exponential dependence similar to that of filopodial-dominated, *anisotropic* spreading fibroblasts [26], underscoring that quantitative image analysis in motility studies can allow us to compare motility modules across cells and even between different cell-types. Anisotropic spreading cells do not, however, exhibit distinct phases of homogenous motility [30], more closely resembling filopodial dominated migration.

Here we present kinematic, molecular, and pharmacological characterizations of the phases of isotropic cell spreading and describe the fundamental motility modules found within these phases: blebbing protrusion and retraction in the early spreading phase, continuous protrusion in the middle phase, and a combination of periodic contraction, continuous protrusion, and quiescence in the late spreading phase. Each motility module is correlated with a unique combination of cell edge dynamics, localization of the cytoskeletal protein VASP, traction-force generation, and response to inhibition of actin polymerization by cytochalasin D. We propose that regulatory pathways give rise to particular combinations of local motility modules, resulting in a global functional phase of the cell.

## Results

### Image Acquisition and Velocity Calculation

**Figure 1:**
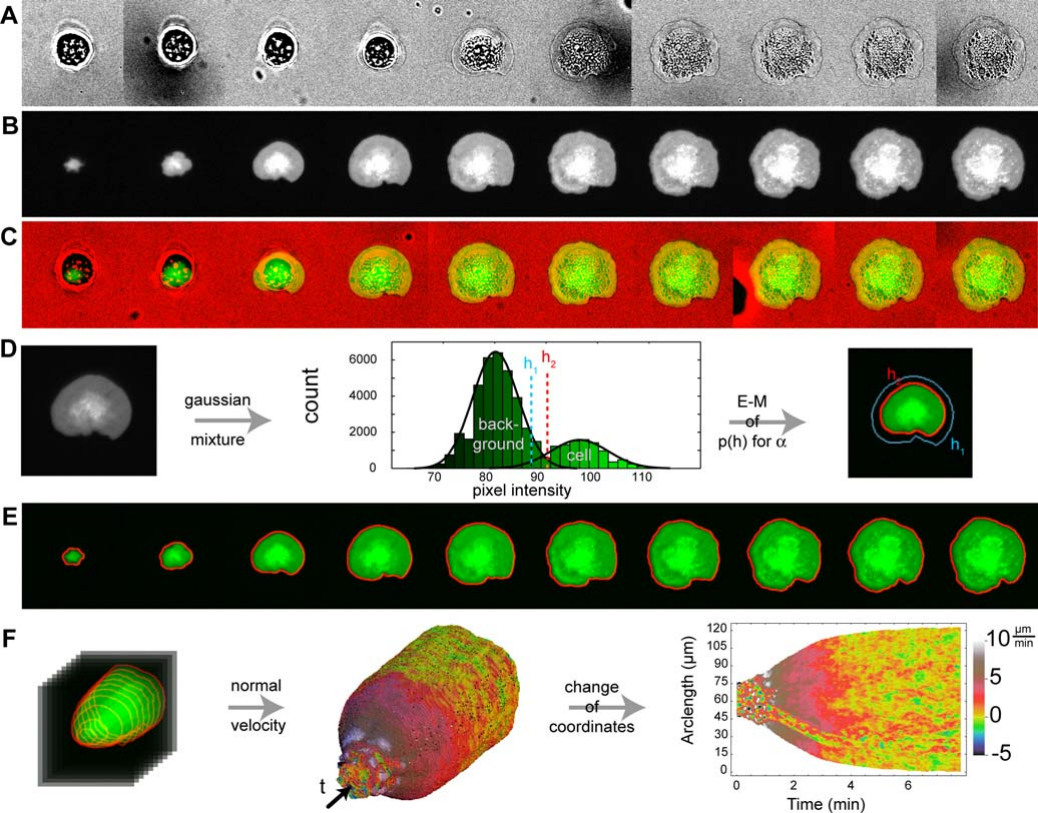
The velocity map encapsulates the kinematics of cell spreading. (A) Bright field sequence of a mouse embryonic fibroblast spreading on a fibronectin coated cover glass (also see Movie S1). Each image is 62 µm high and there is 1 minute between each frame. (B) Same as A except with total internal reflection fluorescence (TIRF) illumination. TIRF reveals only the regions of the cell in closest contact with the surface, allowing for the visualization of edge dynamics at the earliest times. (C) Merge of bright field (red) and TIRF (green) sequences. The cell edge in bright field exactly matches the cell edge in TIRF. (D) Example of Gaussian mixture modeling and expectation-maximization method for image segmentation of a TIRF image (left). A mixture of two Gaussian distributions is used to fit the pixel intensity histogram (middle), where one Gaussian models background pixels and one models pixels corresponding to our fluorescent signal. A threshold is determined by tuning the relative probability that a given pixel intensity belongs to the background or signal distributions (right). The only free parameter in this calculation, performed by a convergent expectation-maximization algorithm, is α, the tightness factor. Two different values of α result in two different values for the threshold, h_1_ and h_2_. (E) Segmentation of the sequence of TIRF images in B for constant α. (F) Projection of the contour sequence into time gives a surface representing edge position as a function of arc-length and time (left). The velocity normal to this surface is then calculated for each point on the surface and encoded by color (middle, also see Movie S2) Finally, by cutting the velocity surface in the time-direction, flattening it, and plotting velocity versus arc-length and time (right), we can observe the spreading velocity of the entire cell edge as it spreads. In order to easily compare the TIRF sequence to the velocity maps, see Movie S2. The cut in the velocity surface occurs at the point corresponding to the right-most point of the cell in the corresponding TIRF image. The positive arc-length direction on the velocity-map corresponds to moving clock-wise around the cell edge in the micrograph. The cell analyzed in this figure corresponds to cell 646 in the database.

We acquired time-lapse micrographs of isotropic spreading cells with both bright field illumination (BF, Fig. 1A) and total internal reflection fluorescence (TIRF, Fig. 1B) using a 20× objective (Movie S1). By exciting only fluorophores within 100 nm of the substrate [31], TIRF revealed membrane dynamics at the earliest spreading times that the cell body usually occludes in bright field imaging (Fig. 1C). Using our Cell Motility Analysis Package (CellMAP, http://cellmap.sourceforge.net/), we calculated the membrane edge position as a function of arc-length and time (Fig. 1D) for each image in the TIRF sequence (Fig. 1E). We then calculated the normal velocity of the cell edge as a function of arc-length and time (Fig. 1F, Movie S2) and performed correlation analyses on the velocity functions. (See materials and methods for details of quantitative analyses.)

### Kinematic Signatures of Spreading Phases

**Figure 2 pone-0003735-g002:**
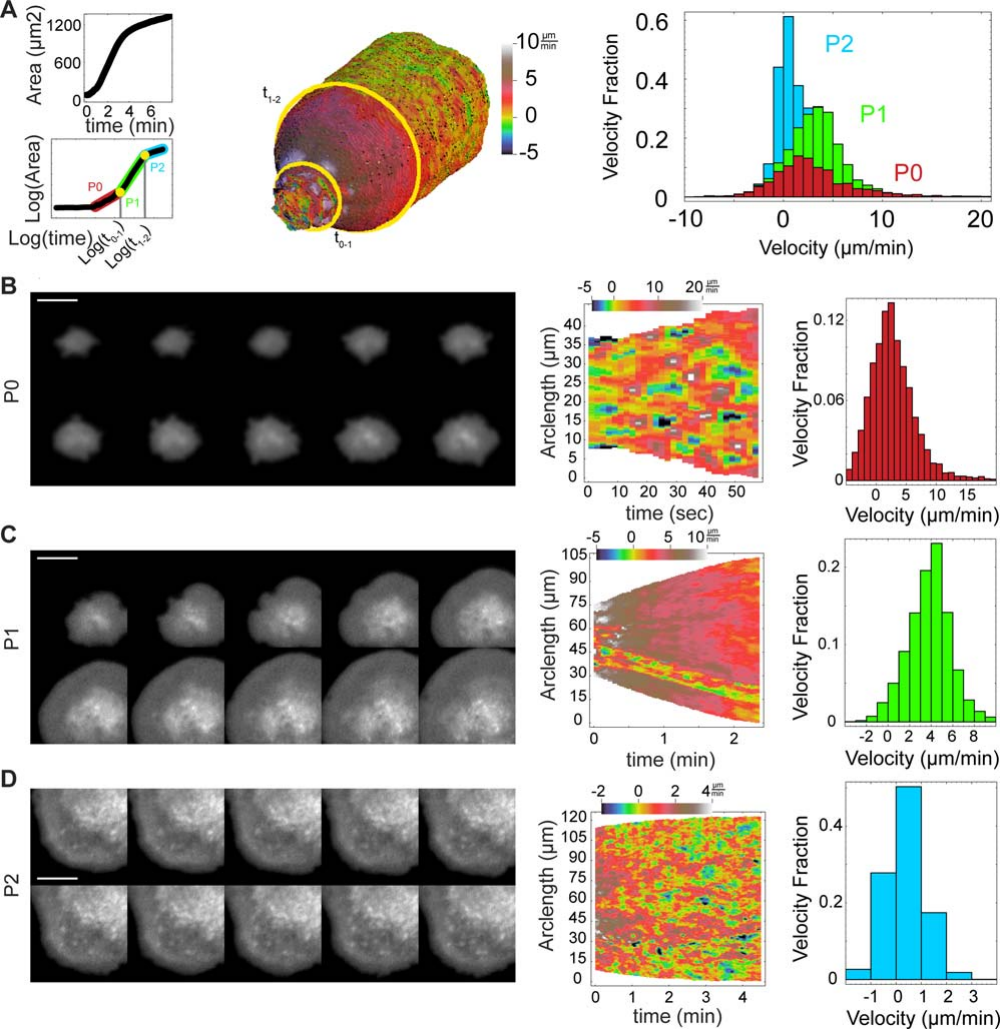
Each spreading phase exhibits a unique combination of motility modules. (A) The time domains of different phases are determined by the best fit of a 3-regime, piecewise function to the logarithm of the area curve (left). These domains are then used to divide the velocity map into different regions (middle). The three phases have distinct normal velocity distributions (right). (B) Phase 0 spreading. Sequence of TIRF images (left) with an interval of 6 seconds. Velocity map (middle). Velocity histogram (right). (C) Phase 1 spreading. Sequence of TIRF images (left) with an interval of 14 seconds. Velocity map (middle) shows continuous protrusion with a line of alternating protrusion and quiescence in a narrow region. Velocity histogram (right). (D) Phase 2 spreading. Sequence of TIRF images (left) with an interval of 14 seconds. Velocity map (middle). Velocity histogram (right). Scale bars represent 10 µm. The cell analyzed corresponds to ID 646 in the database.

We previously observed that isotropic cell spreading can be divided into three phases based on the rate of contact area increase: early spreading (P0), middle spreading (P1), and late spreading (P2) [25]. We hypothesized that each phase represents local cytoskeletal changes (e.g., different motility modules were exhibited) in response to regulatory signals [32]. Fitting the logarithm of cell area vs. time to a piecewise, linear function (Fig 2A), we identified each phase and analyzed edge kinematics in each phase using CellMAP (Fig. 2B–D).

From the velocity maps, we observed that P0 exhibited a regular pattern of small, fast edge protrusions immediately followed by retraction (Fig. 2B), with velocities ranging from −5 µm/min to 20 µm/min. P1, as previously reported [26], was characterized by a uniform and isotropic protrusion of the cell edge (Fig. 2C). Our high temporal resolution (2 seconds between frames) also revealed a 10–15 µm wide, sharply defined region of alternating protrusion and quiescence running through P1 in about 50% of isotropic spreading cells. P2, the late spreading phase (Fig. 2D), exhibited even more variation in edge movements, with many regions of persistent overall protrusion (red regions) mixed with regions of quiescence (green regions) and large retractions (blue regions), with velocities, ranging from −2 to 4 µm/min. While we had previously reported and extensively characterized myosin II dependent periodic lamellipodial contractions in P2 [10], [33], the current analysis represents the first velocity map of an entire cell in P2 in with temporal resolution great enough to resolve periodic contractions. This whole cell analysis reveals that, even in very isotropic cells, periodic contractions coexist with regions of quiescence and even large scale retraction. The membrane retractions resulting from periodic contractions were at or below the limit of resolution of the light microscope and were observed as periodic decreases in the speed of protrusion, different from the other, large scale retractions observed. We hoped, however, that the appropriate quantitative analysis would nevertheless reveal the periodicity in P2 and enable comparison of periodic behavior between P0 and P2.

**Figure 3 pone-0003735-g003:**
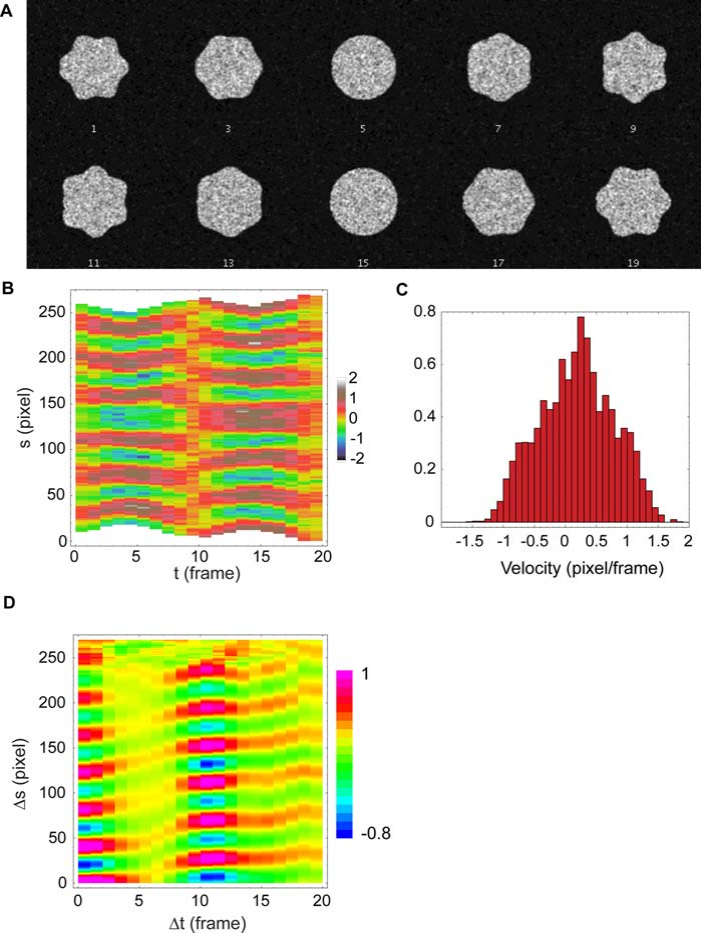
Correlation Plot of Synthetic Data. (A) Synthetic frames mimic the movements of the real cell edge as observed in TIRF. (B) Velocity map of the synthetic data and (C) histogram of measured velocities. (D) Correlation analysis reveals the spatial and temporal extent of regions of motile activity as well as the spatial and temporal spacing between regions of activity.

In order to quantify spatiotemporal periodicities in edge velocity, we calculated a ‘two-point’ auto-correlation function, *c(*Δ*t,*Δ*s)* for the velocity of the edge over space and time. To illustrate, a plot of *c* for simulated data is shown (Fig. 3). Essentially, *c* shows the average pattern of velocity exhibited in a velocity plot. Thus, average spatial and temporal extent of edge activity as well as the spatial and temporal spacing between regions of activity are revealed in the correlation plots, and can quantify patterns of cell edge movement.

**Figure 4 pone-0003735-g004:**
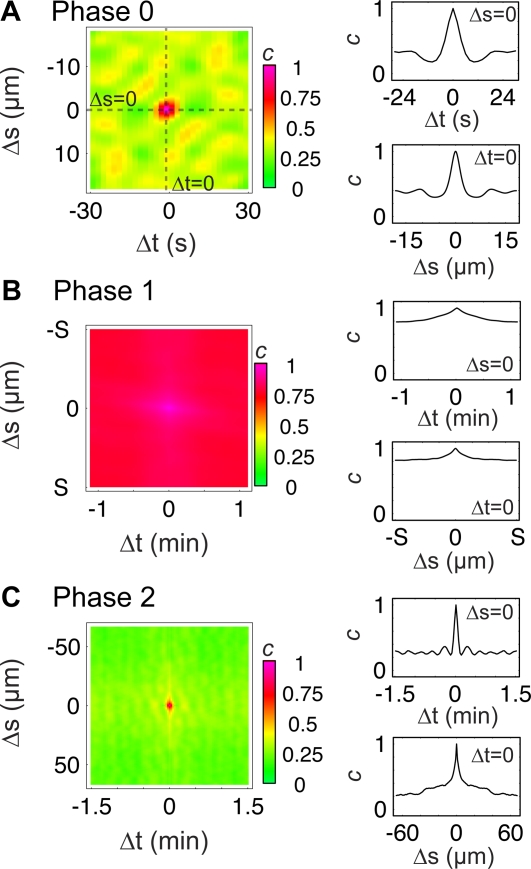
Auto-correlation functions reveal different characteristic lengths and periods in each phase. Two-point correlation function, *c*(Δ*t*,Δ*s*) applied to velocity maps reveal patterns of membrane movement for P0 (A), P1 (B) and P2 (C). Correlation density maps reveal overall patterns (left column) while plots of the Δ*t = *0 or Δ*s = *0 sections (right column) illustrate temporal and spatial features alone. The width of the first peak in *c* around the origin gives the average feature size in time and arc-length for each phase. The distance to the first maximum in the time axis gives the average temporal periodicity of the velocity plot. The distance to the first maximum in the space axis gives the average periodicity in space. Diagonals in the correlation plots reveal lateral propagation of active regions, evident in P0 and P2. Arc-length in P1 is expressed with respect to the maximum arc-length, S, because S(t) is changing rapidly in this phase. Database ID for (A) and (B) is 646, (C) corresponds to ID 625.

Plotting *c* for P0 (Fig. 4A) revealed several features of interest in the velocity plots of the phases of cell spreading. For P0 (Fig. 4A), the average extent of an event was ∼12 seconds in duration (twice the characteristic width of the peak at the origin of *c* along the *t* axis) by ∼6 µm in width (twice the characteristic width of the peak at the origin along the *s* axis). We also found a clear periodicity between protrusion and retractions as seen by repeated peaks and troughs in *c*, both on the *s = *0 axis as well as off-axis. Periodicity in *c* along the *t* axis reveals a period of ∼25 seconds for cycles of edge activity at a given position on the cell. The diagonal, off-axis lines in *c* indicate that activity propagates along the edge with a velocity of ∼0.63 µm/s, consistent with observations in several other cell types [14]. The high positive correlation of P1 in both space and time (Fig. 4B) shows the continuous nature of edge movement in this phase [26], in contrast to the correlation plot for P2 (Fig. 4C). For analysis of P2, we chose a cell whose edge motility was most dominated by periodic contractions, in order to limit our measurements to the periodic contraction motility module and not include regions exhibiting other modules (e.g., quiescence or ruffling). For the periodic contractions motility module, we observed a temporal extent of ∼15 s., temporal oscillations with a period of ∼18 s, and lateral propagation of ∼1.5 µm/s. While these measurements represent average behavior over the entire cell, they are similar to the previous measurements of periodic contractions made in spatially limited regions of the lamellipodium of P2 spreading and migrating cells [10], [25]. In addition, our global analysis reveals that periodic contractions exhibited a spatial correlation of up to ∼30 µm, while previous studies were limited to analyzing smaller (less than 16 µm) segments of the cell edge [33]. Intrigued by these similarities in period and lateral propagation between P2 and P0, we wondered if they reflected similarities in the underlying cytoskeletal organization in these two phases.

### P0 motility modules: non-apoptotic blebbing or filopodia

**Figure 5:**
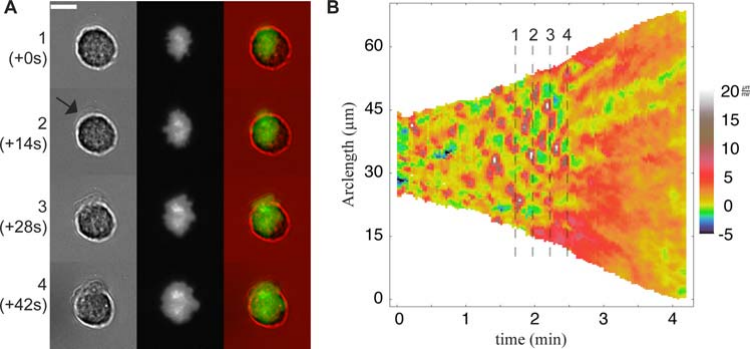
P0 is dominated by periodic blebbing motility module. (A) Bright field (left), TIRF (middle) and merge (right) images of a cell exhibiting blebbing motility (arrow indicates a region of blebbing) during P0 (also see Movie S3). Scale bar is 10 µm. (B) Velocity map of the same cell where the dashed lines indicates the time points represented by the images in (A). The blebs observed in bright field correspond to the regions of patches of protrusion in the velocity map. Cell database ID = 643.

In most cases, membrane movements in P0 could not be observed in bright-field because the cell body occluded the region of surface contact; however, in cases where the cell body was not directly above the site of initial contact, movements in the bright-field images were observed. These movements correlated to those observed in the velocity map and appeared to be extending and retracting membrane blebs (Fig. 5, Movie S3). While blebbing is sometimes a sign of apoptosis, it has also been reported in the early phase of cell spreading [24], mitosis [34], and migration [35]. One mechanism for bleb formation depends upon myosin light chain phosphorylation [36], a mechanism blocked by Rho-kinase inhibitors [37]–[39]. To test if the blebbing we observed in P0 was governed by the same mechanism, we incubated the cells with 20 µM of the Rho-kinase inhibitor Y-27632 for 30 minutes prior to spreading. Under these conditions, bleb formation was inhibited in all cells (n = 125 cells). As in *Dictyostelium*, when blebbing was blocked, P0 was dominated by filopodial motility [40]. Incubation with 20 µM of the myosin light chain kinase (MLCK) inhibitor ML-7 did not inhibit bleb formation (n = 30 cells), adding further evidence that the blebbing motility module in P0 is not apoptotic blebbing [41]. This result suggests that the action of MLCK and Rho-kinase may be spatially segregated in early spreading as they are in fully spread cells [42]. We conclude that in P0, cells exhibit a single protrusive motility module, either non-apoptotic blebbing or filopodium, but not both. Furthermore, while filopodia in P0 are normally associated with anisotropic spreading cells that lack P1 spreading [26], suppressing the blebbing motility module and inducing the filopodia motility module via Rho-kinase inhibition did not inhibit subsequent isotropic P1 spreading. This evidence suggests a regulatory logic in which at least some of the elements regulating the transition from P0 to P1 are ‘up-stream’ of molecules like Rho-kinase that have a direct effect on the motility module expressed by the cell.

Finally, these results show clear differences in motility modules observed between P0 and P2, in spite of similarities in their edge dynamics. First, the periodic contraction motility module is associated with lamellipodial protrusion (Movie S1), not blebbing. Furthermore, periodic contractions are known to be strongly inhibited by ML-7 treatment [10], whereas we found the non-apoptotic blebbing motility module to be insensitive to ML-7. However, we remained curious as to whether the periodicity shared by these two motility modules reflected a functional similarity, a hypothesis we sought to test by measuring the traction forces applied to the substrate during spreading.

### Traction Forces Differ By Phase

**Figure 6:**
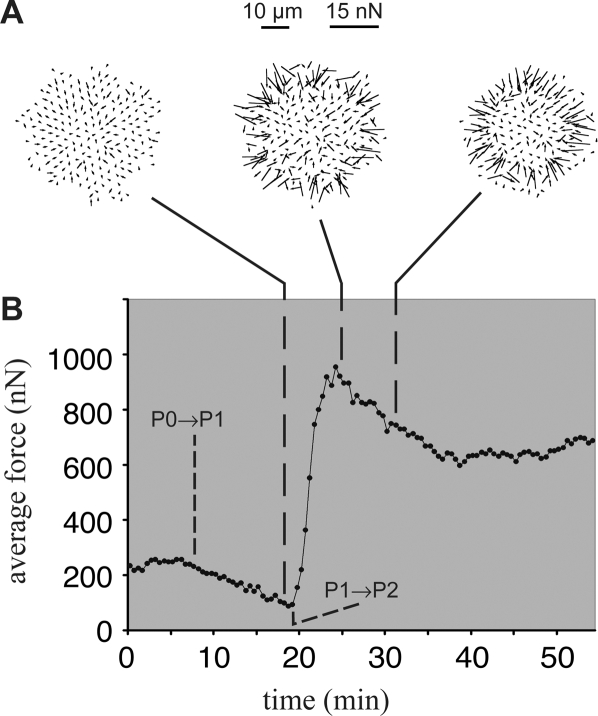
Distinct phases of force generation during spreading. (A) A time-sequence of vector fields shows the direction and magnitude of bending of fibronectin-coated PDMS pillars as a cell exerts force on the substrate during spreading (also see Movie S4). (B) The total applied traction force was calculated for each frame and plotted as a function of time. The sharp increase in traction force at 20 minutes corresponds to the onset of P2, which is followed by a gradual relaxation of force.

In order to observe forces applied to the substrate during cell spreading, we placed cells on polydimethylsiloxane (PDMS) pillars coated with fibronectin (Movie S4). While cells spread more slowly on these pillars, they exhibited the typical isotropic spreading sequence of slow, early adhesion (P0), fast spreading (P1), and late, slower spreading (P2). By measuring the distance pillars were bent (Fig. 6A) we could calculate the total force applied by the cell on the substrate (Fig. 6B). In P0, we observed transient, small displacements of the pillars, indicating that short-lived forces were applied on the substrate in P0 (Movie S4, 0–8 minutes). However, distortion of the pillar images by the cell body prevented precise measurement of forces during P0. Following flattening of the cell body during P1, accurate measurements can be made, and a total force of 100 nN was measured as the cell increased its contact area to 800 µm^2^. The onset of P2 was demarcated by a 5-fold increase in the force exerted on the pillars. Over the next 15 minutes, the force decreased by 40% to a steady state value of about 600 nN. These forces are higher than those reported previously [27] because of higher pillar rigidity [43]. As in P0, transient displacements of individual pillars were observed in P2.

These measurements provide the first quantification of the time course of force generation during cell spreading and support the view that each phase of spreading represents a distinct mechanical state of the cell, with overall forces increasing as the cell spreads. While the total magnitude of force applied by the cell in P2 is much larger than in P0, this could reflect either differences in forces applied by a given edge motility module *or* differences in the ability of the cell, possibly due to reorganization of the cortical cytoskeleton, to transmit and maintain larger traction forces from one side of the cell to the other. In either case, we hypothesize that the oscillatory movement of pillars at the cell edge in both P0 and P2 correlate with the oscillatory edge movements of blebbing and periodic contractions, respectively, suggesting that these two motility modules are functionally similar in their ability to apply periodic, transient force on the substrate. While the precise role of this type of behavior in motility is not well understood, the occurrence of this behavior in both P0 and P2 suggests that it is not accidental and may play an important role, for instance in probing either substrate rigidity or strength of ligand/receptor interactions, or possibly a combination of both.

### VASP localization provides a unique biochemical signature for each motility module

Having observed kinematic, mechanical, and pharmacological differences between the different spreading motility modules, we next sought to find a molecular signature that might serve to distinguish motility modules in fluorescence observation. VASP (NCBI NP_033525), a protein that binds both f-actin and adhesion proteins, is known to stimulate actin polymerization [44], [45] and is localized to the distal tip of the membrane in lamellipodia [46]. During P2 periodic contractions, VASP also localizes to rows of adhesions at the back of the lamellipodium following each contraction [10]. Thus, we hypothesized that the organization of VASP would indicate biophysical and biochemical differences between different motility modules.

**Figure 7:**
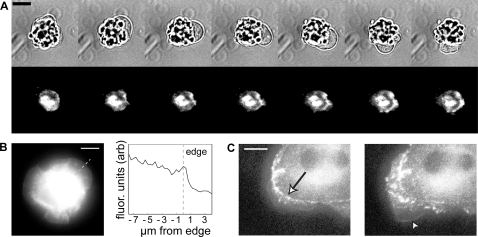
VASP localization distinguishes motility modules. (A) Bright field (top) and TIRF (bottom) time sequence of VASP-GFP transfected cells (see movie S5) reveals that the VASP is not enriched at the tips of P0 blebs during protrusion, instead localizing in adhesions that form at the back of the bleb during bleb protrusion and continuing during bleb retraction. 30 seconds between each frame. (B) During P1, epifluorescence reveals that VASP is concentrated at the leading edge of continuous protrusion, as indicated by a line plot of intensity. The dashed line indicates the region over which the line plot was taken. (C) When the cell enters P2 (also see Movie S6), periodic contractions can occur, with VASP at the tip of the protrusion as well as in rows of adhesions (left, arrow). However, the edge can switch back to a continuous protrusion (C, right), at which point VASP is again localized only at the tip of the protruding region (arrowhead), identical to continuous protrusion in P1. Scale bars represent 10 µm.

Cells transiently transfected with VASP-GFP revealed that VASP was not enriched at the tip of the protruding edge in the blebbing motility module (Fig. 7A, Movie S5), suggesting that VASP-dependent actin polymerization was not directly involved in membrane extension during blebbing. However, both during and after bleb protrusion, an increase in VASP-GFP concentration was observed at the base of each bleb, suggesting that bleb extension and retraction increases local substrate adhesion.

In P1, VASP enrichment was not observed at the membrane edge of regions of the cell exhibiting the continuous protrusion motility module when viewed with our shallow-penetration depth TIRF microscope setup (data not shown). However, observation in epiflourescence revealed that VASP was localized at the tips of the continuous protrusion motility module, though no lasting VASP adhesion sites formed (Fig. 7B). These two observations lead to the hypothesis that VASP is concentrated on the ventral surface of the continuously protruding cell edge, in contrast to VASP localization during periodic contractions.

In P2 regions exhibiting periodic contractions, VASP was deposited in adhesions near the cell edge (Fig. 7C, left), consistent with previous observations [10]. However, when we observed a transition from periodic contractions to continuously protruding membrane, we observed VASP distribution similar to that observed during continuous protrusion in P1 (Fig. 7C, right, Movie S6).

These results confirmed our postulate that VASP distribution was different in each of the spreading motility modules, suggesting that we can differentiate between motility modules by simply observing the static distribution of VASP at the cell edge, dispensing with the need for rapid time-lapse observation. Similarities in VASP adhesion formation during periodic protrusion and retraction in both periodic blebbing and periodic contractions of the lamellipodium further suggested a common function of adhesion formation between these two motility modules, and the transient force generation observed in both modules (see previous section) may stimulate the formation of such adhesions [47]. Inversely, during continuous protrusion, lack of force generation correlates with a lack of VASP adhesion formation, consistent with this mechanism for adhesion formation during spreading.

In addition, we saw that while continuous spreading dominated P1, this motility module was also observed in more limited regions during P2. Furthermore, P2 not only exhibited a mixture of periodic contractions and continuous lamellipodial extension, but also quiescence and large retractions in the form of membrane ruffles (Fig. 7C, Movie S5). These observations were consistent with the combination of different types of edge movement observed in P2 (Fig. 2D), and supported the hypothesis that cells utilize a mixture of motility modules in order to achieve a particular global function (e.g., durotaxis, chemotaxis, phagocytosis).

### Cytochalasin D affects motility modules differently

**Figure 8:**
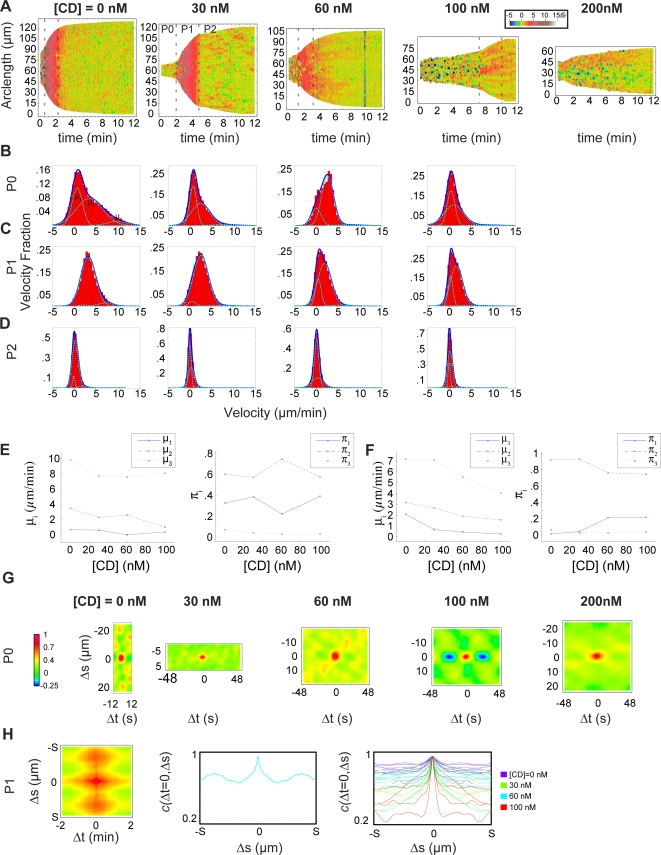
Motility modules depend differently on barbed-end availability. (A) Velocity maps of representative isotropic cells plated following 30 minute incubation with the indicated concentration of CD reveal that motility in P2 is most readily disrupted, followed by P1, with P0 blebbing motility being the least sensitive to CD. Cell ID in database, listed from low to high [CD]: 625, 649 641, 655, 612. (B–D) Velocity histograms for all cells treated with the indicated concentration of CD for P0 (B) P1 (C) and P2 (D). Overlay of a three-component Gaussian mixture model illustrates that different motility modules correspond to different combinations of peaks in the velocity distribution. The *i*th Gaussian component has three parameters, μ*_i_*, σ*_i_*, and π*_i_*, corresponding to the mean, standard deviation, and weight of that component, respectively. CD treatment, while capable of changing the velocity of a particular type of motility (reflected in changes to μ for the corresponding Gaussian component), also alters the probability that a given section of the cell will be in that particular type of motility (reflected in changes to π). (E–F) Summary of the values of μ and π vs. [CD] for the three different Gaussian components in P0 (E) and P1 (F) reveal differences in the dependence on barbed ends for different types of motility. While the velocity of continuous protrusion in P1 decreases with increasing CD (μ_1_ in F, left panel), the fraction of the cell that exhibits continuous protrusion (represented by the increasing π_1_ and decreasing π_2_ π_3_ in F, right panel) decreases as the quiescent fraction increases (π_1_). (G) Correlation maps of P0 vs. [CD] show that CD does not disrupt the spatiotemporal organization of blebbing. Cell ID's are same as in A. (H) 2-D (left) and 1D (middle) correlation plots for a P1 for a cell treated with 60 nM CD exhibited multiple regions of high correlation (Cell ID 631). In general, the extent of high correlation decreases with increasing [CD] (right), indicating that the extent of regions undergoing continuous protrusion is decreasing.

To explore the polymerization complexes involved in the different phases of cell spreading, we treated cells with the actin barbed-end binding toxin, cytochalasin D (CD), over a range of concentrations (0 nM, 30 nM, 60 nM, 100 nM, and 200 nM) for 30 minutes prior to spreading, and analyzed 11, 10, 12, 5, and 8 spreading cells over two trials for each condition, respectively. We generated edge velocity maps for these cells and selected the isotropic spreading cells from the total population for further study (Fig. 8A, Dataset S1&S2). Transitions between phases, defined by changes in the rate of area change (see above), were distinguishable at up to 100 nM of CD, although increased CD concentration disrupted the spatiotemporal organization of motility modules and decreased the final spread area of cells. These results indicated that the mechanism of transition between phases was relatively insensitive to barbed end inhibition by CD, similar to the above finding that altering the motility module of P0 with Rho kinase inhibitor did not affect the P0-P1 transition.

To quantify the effect of CD on different motility modules, we analyzed the distributions of velocities from all cells at a given CD concentration in a specific phase (Fig. 8B–D). Each distribution was fit to a Gaussian mixture model, a linear combination of several Gaussian components, each specified by three parameters; μ_n_ (mean of the nth component), σ_n_ (standard deviation of the nth component), and π_n_ (relative weight of the nth component). Because we had observed that each phase of spreading exhibited a distinct, roughly Gaussian velocity distribution (Fig. 2), we hypothesized that each component of the mixture model would represent a particular underlying motility module-quiescence, bleb protrusion, bleb retraction, or continuous extension - and μ, σ, and π characterized the edge kinematics of each mechanism.

We used a three-component mixture model to describe P0, reflecting that this spreading phase was comprised of a combination of barbed end independent motility (blebbing), a barbed end dependent motility, and quiescence. The high-velocity Gaussian component (n = 3) corresponded to the high-velocity bleb protrusion events, and the speed of these protrusions changed by a relatively small amount across the range of CD treatments, from 10 µm/min to 8 µm/min (Fig. 8B&E). The low-velocity Gaussian component in P0 (n = 2) revealed a population of protrusion events with a velocity distribution centered at 4 µm/min under control conditions that shifted to 1 µm/min at 100 nM CD. The final Gaussian component represented the quiescent regions of the cell whose velocity remained unchanged with CD treatment (n = 1).

We also used three-component Gaussian mixture model for the velocities in P1. One component (n = 1) modeled the quiescent regions of the cell for each treatment, with the exception of control cells where there were few quiescent regions in P1. The other two Gaussian components (n = 2 & n = 3) modeled the distribution of velocities in continuously protruding regions of the cell. The mean velocities of both components of the continuous protrusion motility module decreased at higher CD concentrations, although the most dramatic decrease was in the fraction of the edge exhibiting these high velocities. Indeed, as CD treatment increased, the probability of a given part of the cell being quiescent (π_1_) increased. There was an abrupt shift at 60 nM CD (Fig. 8C,F), and we hypothesize that at this concentration of CD a pool of excess barbed-ends was finally eliminated by CD barbed-end capping. Furthermore, this analysis as well as direct observation of velocity maps show that a given part of the cell exhibited *either* the continuous protrusion motility module *or* quiescence supported our hypothesis that each motility module represents a stable and discrete state of cytoskeleton organization, as opposed to a continuum of intermediary states.

To further quantify the disruption of the organization of motility modules by CD, we applied correlation analyses. Analysis of P0 (Fig. 8B) revealed the least disruption of spatiotemporal patterning–the blebbing motility module was essentially unchanged by increasing concentrations of CD (Fig. 8G). P1 motility, however, underwent a substantial shift in organization; while cells continued to exhibit highly correlated spatial regions of persistent activity, correlation maps revealed cells that exhibited multiple isolated spatial domains of high correlation (Fig. 8H, left, middle). In general, the spatial extent of correlation decreased as CD was added (Fig. 8H, right). These results indicated that while the biochemical and biophysical conditions in the cell during P1 were favorable for continuous protrusion, the presence of CD decreased the probability for continuous protrusion of the cytoskeleton in a given local region of the cell due to a decrease in the barbed-end availability.

This data provided a general kinematic fingerprint for the periodic blebbing and continuous protrusion motility modules while showing that each motility module exhibited a different dependence to barbed-end availability. Periodic blebbing exhibited a small, high velocity population (high μ_3_, low π_3_) combined with a lower velocity CD sensitive population (μ_2_) and quiescent regions (μ_1_) while the periodicity of blebbing was insensitive to CD. The high velocity populations (μ_2_ and μ_3_) in P1 were more sensitive to CD and showed a marked increase in quiescence at 60 nM CD (π_1_). This data, combined with the observation of domains of continuous protrusion, indicated that continuous protrusion and quiescence were discrete states, and that a continuum of states between protrusion and quiescence does not exist.

### Motility after P2

**Figure 9:**
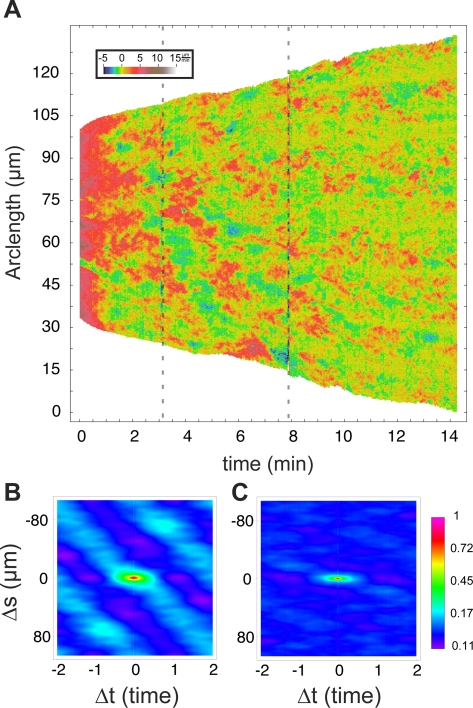
Laterally propagating domains of periodic contractions. (A) After entering P2, some cells exhibit a lateral propagation of regions exhibiting periodic contractions. This large-scale lateral propagation is seen by correlation analysis (B) performed in the region of the velocity map indicated by the dashed grey lines. After 8 minutes, the speed of lateral propagation decreases (C). The suppression of lateral propagation may be an important step in establishing polarization.

During polarization and migration, the cell is biased to exhibit net-protrusive motility modules in one region while motility modules giving a net-retraction occurred in a diametrically opposed region. In contrast, isotropic cell spreading is not a polarized process. In high-resolution time-sequences of P2 fibroblasts (1 second between frames, 0.11 µm pixel size), regions exhibiting periodic contractions were observed to move laterally at an average rate of 0.38 µm/s and in some cases traveled around the entire circumference of the cell, moving as far as 90–100 µm (Fig. 9 A, B). However, as time progressed, the rate and coherence of this propagation greatly decreased (Fig. 9 C). This suppression of lateral propagation of edge activity could represent an important step in the development of polarized motility in a limited region, as was recently observed in formation of immune synapse. In this case, the kinase PKCθ was required for the maintenance of polarity and migration in T-cells–in cells lacking PKCθ, the lateral propagation of activity was unchecked, preventing cells from forming a stable cell front and moving in a directed manner [48]. Suppression of propagation of motility modules can would localize activity along the cell edge, and could be a general mechanism of cell polarization, or phase 3 (P3) of spreading.

## Discussion

Isotropic cell spreading is a process during which a cell exhibits a small number of motility modules in coordination with two sharp transitions in global behavior (summarized in table 1). Detailed characterization of the motility modules during spreading (summarized in table 2) reveals that they are similar to those observed during more general motile phenomena. For instance, cells can exhibit membrane blebbing during mitosis [34], development [49], [50], and cancer cell movement [35], [51]. Lamellipodial dominated motility very similar to P1 continuous spreading has been observed in post-mitotic cell spreading [30] and keratocyte migration [52], as well as in tumor-derived epithelial cell lines exposed to epidermal growth factor, which undergo a two minute long period of rapid actin polymerization [53]. Furthermore, we showed previously that periodic contractions similar to P2 were present in migrating fibroblasts and endothelial cells, and is one of the most fully understood motility modules in migrating cells [10]. Thus, the quantitative characterization of spreading motility modules can provide an important aid in comparing mathematical models of motility [54] to experiment. In addition, we have begun the development of a set of kinematic (Fig. 1–5&9), mechanical (Fig. 6) and molecular (Fig. 7,8) fingerprints for different motility modules that, combined with an understanding of the molecular machines that contribute to those motility modules [33], [38], [55], will allow us to probe the molecular-level function of a given perturbation based on our relatively low resolution, high-throughput, quantitative spreading assay. Such an approach could provide fast, highly interpretable functional screens for chemical libraries, siRNA libraries [27], or tumor cells.

**Table 1:** Correspondence between spreading phases and motility modules.

Phase of Isotropic Spreading	P0	P1	P2
**Motility Modules Observed**	•Periodic Blebs	•Continuous Protrusion	•Protrusion w/Periodic Contractions
	•Filopodia	•Quiescence	•Continuous Protrusion
	•Quiescence		•Ruffling
			•Filopodia
			•Quiescence

Each phase of isotropic spreading is associated with a limited number of motility modules. Here we summarize the different motility modules that we have observed in each phase.

**Table 2:** Properties of Motility Modules.

Motility Module	Temporal Periodicity	Propagation Speed	Traction Force	VASP Localization	Inhibited by:
Healing Blebs	∼25 seconds	∼0.6 µm/s	Low	Adhesions at base of bleb	RhoK inhibitor
Continuous Protrusion	None	None	Low	Tip of protrusion	High Cytochalasin D
ProtrusionsPeriodic Contractions	∼20 seconds	∼1.5 µm/s	High	Tip and back of lamellipodium	Cytochalasin D, MLCK inhibitors

Each motility module can be defined by a unique combination of biophysical, molecular, and pharmacological characteristics.

Spreading assays owe their interpretability to the homogenous nature of motility during isotropic cell spreading. Sharp temporal transitions occur between phases, and we have shown that this transition is the result of a rapid shift in the combination of motility modules exhibited. In P0, we observed that cells exhibit a filopodial motility module or a blebbing motility module, but not both, and that the phenotype was dependent on Rho-kinase activity. A sharp transition from blebbing in P0 to the continuous protrusion motility in P1 was observed, with very little overlap between blebbing and continuous protrusion. However, in P1, while many untreated cells contained small regions that did not exhibit continuous protrusion, these regions had sharp boundaries and did not alter the behavior of the continuous protrusion proceeding on either side. Upon cytochalasin D treatment, continuous protrusion in P1 decreased in edge protrusion speed and regions of quiescence were formed. However, the boundary between the regions of continuous protrusion and quiescence regions were generally very sharp, with certain regions exhibiting continuous protrusion while other regions remained quiescent. In P2, we found that discrete regions of the cell exhibit protrusive motility modules, and that these regions can maintain coherence over long times and distances (Fig. 9). Protrusive motility in P2 can consist of periodic contractions, continuous protrusion, and large membrane ruffles, but each maintains the distinct molecular identity of a motility module (Fig. 7). Together, these observations support our hypothesis that each motility module represents a dynamic state of the cytoskeleton that is stable over a range of biochemical and biophysical conditions, but that when this range is exceeded, a transition to a different motility module occurs. In future work, the velocity map could be replaced by a *module map*, in which velocity information in the velocity map would be replaced simply by an indication of the motility module exhibited at that particular location and time on the cell edge. This high level of data reduction and abstraction would be highly advantageous for statistical analyses of cytoskeletal behavior.

The hypothesis of discrete motility modules is consistent with our proposed model of hierarchical motility regulation [32]. At the lowest level of the hierarchy are actin and proteins that directly modify actin dynamics (e.g., actin polymerization factors such as VASP, myosin motors). Particular complexes of these molecules give rise to stable states of actin dynamics, resulting in a stereotypic kinematic signature of the cell edge. At the higher levels of the hierarchy are molecules that lead to global organizational changes such as those observed in transitions between spreading phases and those factors that control the generation of traction forces. Candidate molecules are the Rho family GTPases [56] or calcium signals induced by integration of chemical or mechanical signals [57], both of which exhibit abrupt changes in activity or concentration in response to cell spreading and may be involved in the global regulation of spreading phases. However, motility modules can also be organized locally, as occurs during polarization and migration when lamellipodial contractions and ruffling are switched on only in the protruding regions of the cell. Such modular regulation has been observed in the switching between migrational modes in neurons [58], [59], amoeba [40], immune synapses [60], tumor cells [35], [59], [61], and Dictyostelium [62]. That these many different cell types share many motility modules in common suggests that these stable states of cytoskeleton dynamics are relatively conserved, and that differences between cells is primarily achieved at the higher levels of the regulatory hierarchy. In this paper, we have provided the biophysical and biochemical characterization of motility modules required to test this hypothesis.

Generation of traction forces on the substrate was shown previously to be primarily dependent upon myosin II A and B isoforms [27]. We find that relatively little force is generated during phase 1, which is consistent with the slow rate of actin retrograde flow [10], [26] and the ability of cells to spread in this mode with no requirement for further integrin binding [26]. The rapid rise in force exposes a major change in cell state from phase 1 to phase 2. We postulate that this transition reflects a global activation of myosin through a RhoA kinase pathway, although further experimentation is required to differentiate such a biochemical hypothesis from a more biophysical mechanism such as a sudden increase in membrane tension as the cell spreads. Additionally, based on our pharmacological perturbations, we found intriguing difference between the Rho-kinase and MLCK myosin II pathways in P0. However, none of these cells exhibited an impaired ability to initiate P1; indeed, cells treated with impaired or ablated myosin II activity were able to initiate P1 [33]. We propose that while myosin force generation in P0 plays a role in promoting early adhesion formation (Fig. 7A) and could be of functional importance under conditions where fluid flow shears the cell, integrin signaling alone is sufficient to initiate P1.

The property of continuous protrusion during P1 represents an experimental system that can be used to test biophysical models for how actin generates edge protrusion (see Appendix S1). In general, testing the predictions of models against *in vivo* experiments is difficult because cells rarely undergo large scale, steady-state protrusion. Therefore, most of the experimental constraints on these models are based on *in vitro* data. However, during P1 continuous spreading, the actin cytoskeleton is in a spatially homogenous protrusive steady-state. These protrusions are independent of substrate adhesion [26], [63], exhibit much less traction force on the substrate, and are independent of myosin II activity [33]. Thus, P1 continuous spreading represents a motility module in which actin polymerization against the membrane is the dominant motile event, making P1 spreading an ideal cell state on which to test the predictions of mathematical models of cytoskeletal protrusion.

While the study of the continuous protrusion motility module can experimentally isolate actin mechanochemistry, the lateral propagation of activity over a long-distance in both P0 blebbing and P2 periodic contractions make these systems ideal for studying the biophysical nature of these propagations. Theoretical models have suggested that lateral propagation can be generally explained by a combination of myosin motors and polymerization-stimulating membrane proteins aggregating in regions of convex membrane curvature [64]. Our results suggest that this model may not apply in the case of lateral propagation of blebbing motility, since high concentrations of CD would likely disrupt polymerization from a membrane bound protein but do not disrupt the lateral propagation of blebbing in P0. Interestingly, myosin II is required for normal function of both of these motility modules, though blebbing motility depends on Rho kinase and not the myosin light chain kinase that was previously shown to disrupt periodic lamellipodial contractions [10]. At present however, we do not understand the cause of lateral propagation in P0 blebbing.

In a recent editorial on the state of systems biology, George Church asks how the rest of biology can “reach the enviable status of bioinformatics and crystallography?” and suggests that sharing data is a crucial step towards achieving this goal [65]. All data for the cells analyzed in this paper, along with their corresponding two-dimensional velocity maps and the open source software CellMAP, are available at http://cellmap.sourceforge.net. In the spirit of projects such as the Open Microscopy Project (http://www.openmicroscopy.org), we hope that making our data and software freely available will provide a model for a collaborative future in the field of cell motility and guide the way to a systematic approach for storing and distributing cell image data, such as already exists in the fields of protein biophysics.

## Materials and Methods

### Cell Culture and Sample Preparation

We utilized immortalized mouse embryonic fibroblast cells grown in Dulbecco's Modified Eagle Medium (DMEM) supplemented with 10% fetal bovine serum, 100 IU/ml of Penicillin-Streptomycin, 2 µM of L-Glutamine, and 2 µM of HEPES. Cultures were maintained at 37°C in a 5% CO_2_ incubator and sub-cultured prior to reaching 70% confluence. Culture reagents were purchased from Gibco-Invitrogen.

Spreading assays were performed as previously described [26]. Briefly, cells were grown to 70% confluence, trypsinized quickly washed with soybean trypsin inhibitor, centrifuged, and resuspended in phenol red and serum-free DMEM (Gibco-Invitrogen). Next, cells were incubated for 20 minutes at 37°C, followed by a second 20 minute incubation with 5 µM calcein red-orange-AM (Molecular Probes). Cells were then centrifuged and resuspended prior to plating.

Cover glasses were washed 2 h. in 20% nitric acid and then silanized by exposure to gaseous 1,1,1,3,3,3-Hexamethyldisilazane (Sigma). We created a well on each cover glass using silicone isolators (Grace Bio-Labs, Inc.) and coated the silanized cover glass with 600 µL of a 10 µg/ml human plasma full-length pure fibronectin (Sigma) solution for 1 hour at 37°C.

Cytochalasin D, Y-27632, and ML-7 were added to the cell suspension prior to plating for the time and concentration indicated. In all cases drug concentrations were maintained throughout the experiment.

### Microscopy

Total Internal Reflection Fluorescence (TIRF) and bright-field time-lapse microscopy were performed as previously described [26]. Cells were imaged with a 20×, 0.95NA water immersion objective (Olympus) on an Olympus BX-51 upright microscope. A custom stage was positioned above a stationary quartz dove prism (Edmund Scientific). Index of refraction matching immersion oil was added to the cover glass-prism interface. TIRF excitation was achieved using the 568 nm emission from an argon-ion laser (Melles Griot) and passed through the prism at an angle of incidence at the cover glass-water interface of less than the critical angle to achieve total reflection, generating an evanescent wave approximately 100 nanometers into the sample medium. For bright field, the prism precluded the use of a condenser. A Cool Snap FX cooled CCD camera (Roper Scientific) controlled by SimplePCI (Compix Inc.) software was used to record the time-lapse micrographs.

### Cell Motility Analysis Platform (CellMAP)

CellMAP is a suite of Matlab, Mathematica, and C/C++/ObjC command line programs designed to for the quantitative analysis of cell motility [14], [28]. Input to CellMAP is any high contrast, time-lapse fluorescence sequence of a single cell whose boundary lies entirely within every frame of the sequence (Fig. 1b). Outputs include (but are not limited to): arc-length-parameterized contours for each frame in the sequence, the normal velocity of the cell edge as a function of space and time, the area of the cell as a function of time, and a cross-correlation plot for the normal velocity as a function of arc-length and time.

### Segmentation and Normal Velocity Calculation (noVel)

The problem of cell segmentation for a time-lapse sequence of TIRF images can be stated as follows: at each location *i* in a given frame we observe an image pixel *h_i_* and wish to infer the underlying scene pixel *q_i_*, where *q_i_* ∈{+,−} for pixels inside and outside the cell, respectively. We work under a Gaussian noise model where, given the *q_i_*′s, the *h_i_*′s are centered about class means *μ*_±_ with class standard deviations *σ*_±_, all of which must be inferred from the data. We assume all pixels are independent and identically distributed, with no spatial coupling between class values at neighboring scene locations.

For each frame in the sequence we fit a two-component Gaussian mixture model of the form
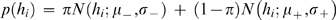
(1)to the distribution of pixel intensities (Fig. 1D) using Expectation Maximization, an iterative, unsupervised learning algorithm [66]. With the *q_i_*′s, *μ*_±_ and *σ*_±_ now determined, a time-dependent intensity threshold 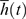 that satisfies
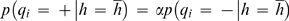
(2)(for a user-specified *α*) is calculated. The inside of the cell is segmented from the background and the resulting cell boundary Γ(*s,t*) is parameterized by arc-length *s*(*t*).

The normal velocity of each point on Γ(*s,t*) is calculated from gradients of the image data *h*(**x**(*s*,*t*),*t*) as
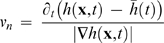
(3)This is equivalent to the kinematic boundary condition in fluid dynamics and a simpler case of the velocity inference problem often addressed by optical flow methods [67], [68]. The normal velocity as a function of arc-length and time is displayed in a color-coded plot.

There are several advantages of the above method over previously employed techniques [12], [26]. Firstly, CellMAP automates cell segmentation, allowing for only one user-controlled parameter, *α*, which has a mathematically principled and highly interpretable origin: a pixel of intensity 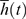 is *α* times as likely to have been drawn from the foreground class than from the background class; *α* controls the “tightness” of the contour. This parameter applies across all frames, removing uncertainties and fluctuations introduced by manual thresholding of individual frames.

In addition, arc-length parameterization of the cell contour enables one to analyze the non-convex morphologies encountered in early spreading and highly polarized cells in which polar coordinate descriptions fail due to multi-valued *r*(*θ*,*t*) relations.

Finally, optical flow velocity calculation provides an accurate measure of the normal velocity for all points on the cell boundary. It should be noted that in highly anisotropic cells, the normal direction often differs dramatically from the radial direction; optical flow accurately captures normal velocity information for such cells via image gradients in a computationally-efficient manner without the need to explicitly construct local normal vectors.

### Correlation Analysis

We employed a two-point correlation function to quantify the spatiotemporal patterns of protrusions and retractions in a cell. The discrete form of the correlation function is given by
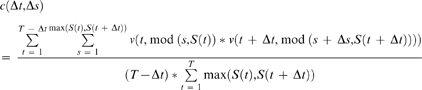
(4)where *v(t,s)* is the mean-subtracted membrane normal velocity as a function of arc-length *s* and time *t*, Δ*t* is the lag in time, Δ*s* is the lag in space, *T* is the maximum length of time in *v(t,s)* and *S(t)* is the total arc-length in *v(t,s)* as a function of time. The modulus function, mod(*x,*X), is used in the spatial coordinate to establish periodic boundary conditions in the spatial dimension.

This correlation function compensates for changes in the total contour length as the cell increases and decreases in area. However, as it involves several explicit loops, it is also inherently computationally expensive. Therefore, we made use of the Wiener-Khinchin theorem that states that the auto-correlation function is equivalent to the inverse Fourier transform of the absolute value squared of the Fourier transform of a function. This approach was orders of magnitude faster due to the speed gained through using fast Fourier transform (FFT) algorithms on our discrete data. However, as FFT requires rectangular matrices as input, we sampled, via linear interpolation, a constant number of points along the contour with respect to time. The drawback of this approach is that one loses the ability to measure the spatial-lag in terms of arc-length. For P0 and P2, where the contour-length changes very little, we assigned the total length of the spatial-lag axis as the average arc-length of the cell in that phase. Comparison of results between the two-point and FFT based correlation functions were practically identical. For P1, where the total arc-length changes dramatically, length units are somewhat arbitrary and were simply scaled between 0 and 1. In all cases, the magnitude of the correlation function was normalized for unity at zero-lag.

### Force measurement with pillars

The preparation, characterization, and measurement of deflection of polydimethylsiloxane (PDMS) pillars are described in detail elsewhere [43], [69]. The dimension of the PDMS pillars used here was 2 µm in diameter, 3 µm center-to-center, and 6 µm in height. The spring constant of the pillars was 13.7 nN/µm. Fibronectin coating of pillars was achieved by immersing pillars in 10 µg/ml of fibronectin solution for 2 hr at 37°C followed by washing with DPBS. Then, cells were plated on the pillars to allow spreading in the presence of serum. Time-lapse images of the pillars tips were captured with a LUCPIanFI 40×/0.60 air objective in bright-field mode on an IX71 Olympus inverted microscope. A multiple-particle tracking program [43], [69] was used to analyze the displacement of the pillars. Systematic error was subtracted in the calculation of the pillar deflection.

## Supporting Information

Appendix S1 From an analysis of membrane edge velocities, we estimate the kinetic constants of actin polymerization as a function of Cytochalasin D.
(0.05 MB DOC)

Dataset S1 Velocity vs. [CD] Part I. Part I of II. Velocity maps of isotropic cells used in the CD spreading dependence studies. The numbers above each plot indicates the cell ID # in our database. The data for these velocity plots, area vs. time curves, and sample algorithms for visualizing data are all accessible through http://cellmap.cellmotion.org/.
(11.80 MB TIF)

Dataset S2 Velocity vs. [CD] Part II. Part II of II. Velocity maps of isotropic cells used in the CD spreading dependence studies. The numbers above each plot indicates the cell ID # in our database. The data for these velocity plots, area vs. time curves, and sample algorithms for visualizing data are all accessible through http://cellmap.cellmotion.org/.
(4.99 MB TIF)

Movie S1 Cell Spreading (Re: Figure 1). A time-lapse of bright field (red), TIRF (green) micrographs and their overlay (right) shows an immortalized mouse embryonic fibroblast spreading onto a fibronectin coated cover glass.
(6.25 MB MOV)

Movie S2 Velocity Map Analysis (Re: Figure 1). Our algorithms calculate the contour position and the velocity in the direction of the normal to the contour during spreading. The TIRF sequence of an isotropic spreading cell with the contour position overlaid illustrates our technique (left). Each point on the contour is colored to represent the velocity in the direction of the normal to the cell edge at that point (see Fig. 2 for color scale). By stretching out and placing each contour in sequence, we generate the basic unit of our quantitative analysis of cell motility, the velocity map (right). The vertical bars indicate the progression of time. In order to easily compare the TIRF sequence to the velocity maps, keep in mind that the cut in the velocity surface occurs at the point corresponding to the right-most point of the cell in the corresponding TIRF image, and moving in the positive arc-length direction on the velocity-map corresponds to moving clock-wise around the cell edge in the micrograph.
(5.12 MB MOV)

Movie S3 P0 Blebbing (Re: Figure 5). Bright field (left) TIRF (center) and merged (right) images of an isotropic spreading immortalized mouse embryonic fibroblast cell exhibiting P0 blebbing motility. Scale bar represents 5 µm. Frames were collected every two seconds and the display rate is 30 frames per second.
(4.07 MB MOV)

Movie S4 Spreading Traction Forces (Re: Figure 6). An array of flexible pillars coated with 10 µg/ml of fibronectin is observed as a mouse embryonic fibroblast spreads onto the surface. Frames were collected every 30 seconds and the display rate is 10 frames per second.
(0.95 MB MOV)

Movie S5 VASP Recruitment During Blebbing (Re: Figure 7A). TIRF time-lapse of bright field (left) GFP-VASP (center), and merge (right) of a cell in P0. It is observed that VASP enrichment in surface adhesions form during bleb protrusion and retraction. Scale bar represents 10 µm.
(0.33 MB MOV)

Movie S6 Multiple Motility Modules in P2 (Re: Figure 7C). TIRF time-lapse of GFP-VASP (left), DIC (center), and merge (right) of a cell in P2. Four motility modules, periodic contractions, continuous protrusion, ruffling, and quiescence, can all be observed. Scale bar represents 10 µm.
(3.11 MB MOV)

## References

[1] Dustin ML, Allen PM, Shaw AS (2001). Environmental control of immunological synapse formation and duration.. Trends in Immunology.

[2] Friedl P, den Boer AT, Gunzer M (2005). TUNING IMMUNE RESPONSES: DIVERSITY AND ADAPTATION OF THE IMMUNOLOGICAL SYNAPSE.. Nature Reviews Immunology.

[3] Singer AJ, Clark RA (1999). Cutaneous wound healing.. N Engl J Med.

[4] Baum B (2004). Animal Development: Crowd Control.. Current Biology.

[5] Winklbauer R, Selchow A (1992). Motile behavior and protrusive activity of migratory mesoderm cells from the Xenopus gastrula.. Dev Biol.

[6] Ninov N, Chiarelli DA, Martin-Blanco E (2007). Extrinsic and intrinsic mechanisms directing epithelial cell sheet replacement during Drosophila metamorphosis.. Development.

[7] Condeelis J, Singer RH, Segall JE (2005). THE GREAT ESCAPE: When Cancer Cells Hijack the Genes for Chemotaxis and Motility.. Annual Review of Cell and Developmental Biology.

[8] Kaplan RN, Riba RD, Zacharoulis S, Bramley AH, Vincent L (2005). VEGFR1-positive haematopoietic bone marrow progenitors initiate the pre-metastatic niche.. Nature.

[9] Pollard TD (2003). The cytoskeleton, cellular motility and the reductionist agenda.. Nature.

[10] Giannone G, Dubin-Thaler BJ, Dobereiner HG, Kieffer N, Bresnick AR (2004). Periodic lamellipodial contractions correlate with rearward actin waves.. Cell.

[11] Medeiros NA, Burnette DT, Forscher P (2006). Myosin II functions in actin-bundle turnover in neuronal growth cones.. Nat Cell Biol.

[12] Machacek M, Danuser G (2006). Morphodynamic profiling of protrusion phenotypes.. Biophys J.

[13] Silva HS, Martins ML, Vilela MJ, Jaeger R, Kachar B (2006). 1/f ruffle oscillations in plasma membranes of amphibian epithelial cells under normal and inverted gravitational orientations.. Phys Rev E Stat Nonlin Soft Matter Phys.

[14] Döbereiner HG, Dubin-Thaler BJ, Hofman JM, Xenias HS, Sims TN (2006). Lateral membrane waves constitute a universal dynamic pattern of motile cells.. Phys Rev Lett.

[15] Heo WD, Meyer T (2003). Switch-of-function mutants based on morphology classification of Ras superfamily small GTPases.. Cell.

[16] Vasiliev JM (1982). Spreading and locomotion of tissue cells: factors controlling the distribution of pseudopodia.. Philos Trans R Soc Lond B Biol Sci.

[17] Bardsley WG, Aplin JD (1983). Kinetic analysis of cell spreading. I. Theory and modelling of curves.. J Cell Sci.

[18] Price LS, Leng J, Schwartz MA, Bokoch GM (1998). Activation of Rac and Cdc42 by integrins mediates cell spreading.. Mol Biol Cell.

[19] de Hoog CL, Foster LJ, Mann M (2004). RNA and RNA binding proteins participate in early stages of cell spreading through spreading initiation centers.. Cell.

[20] Adamsky K, Schilling J, Garwood J, Faissner A, Peles E (2001). Glial tumor cell adhesion is mediated by binding of the FNIII domain of receptor protein tyrosine phosphatase beta (RPTPbeta) to tenascin C. Oncogene.

[21] Tamura M, Gu J, Matsumoto K, Aota S, Parsons R (1998). Inhibition of cell migration, spreading, and focal adhesions by tumor suppressor PTEN.. Science.

[22] von Wichert G, Jiang G, Kostic A, De Vos K, Sap J (2003). RPTP-alpha acts as a transducer of mechanical force on alphav/beta3-integrin-cytoskeleton linkages.. J Cell Biol.

[23] Bliokh Zh L, Smolianinov VV (1977). [Kinetics of fibroblast spreading].. Biofizika.

[24] Bereiter-Hahn J, Luck M, Miebach T, Stelzer HK, Voth M (1990). Spreading of trypsinized cells: cytoskeletal dynamics and energy requirements.. J Cell Sci.

[25] Döbereiner HG, Dubin-Thaler B, Giannone G, Xenias HS, Sheetz MP (2004). Dynamic phase transitions in cell spreading.. Phys Rev Lett.

[26] Dubin-Thaler BJ, Giannone G, Dobereiner HG, Sheetz MP (2004). Nanometer analysis of cell spreading on matrix-coated surfaces reveals two distinct cell states and STEPs.. Biophys J.

[27] Cai Y, Biais N, Giannone G, Tanase M, Ladoux B (2006). Nonmuscle Myosin IIA-dependent Force Inhibits Cell Spreading and Drives F-actin Flow. Biophys J.

[28] Ada-Nguema AS, Xenias H, Sheetz MP, Keely PJ (2006). The small GTPase R-Ras regulates organization of actin and drives membrane protrusions through the activity of PLCepsilon.. J Cell Sci.

[29] Betz T, Lim D, Kas JA (2006). Neuronal Growth: A Bistable Stochastic Process.. Physical Review Letters.

[30] Cuvelier D, Thery M, Chu YS, Dufour S, Thiery JP (2007). The universal dynamics of cell spreading.. Curr Biol.

[31] Axelrod D, Thompson NL, Burghardt TP (1983). Total internal inflection fluorescent microscopy.. J Microsc.

[32] Döbereiner HG, Dubin-Thaler BJ, Giannone G, Sheetz MP (2005). Force sensing and generation in cell phases: analyses of complex functions.. J Appl Physiol.

[33] Giannone G, Dubin-Thaler BJ, Rossier O, Cai Y, Chaga O (2007). Lamellipodial actin mechanically links Myosin activity with adhesion-site formation.. Cell.

[34] Boss J (1955). Mitosis in cultures of newt tissues. IV. The cell surface in late anaphase and the movements of ribonucleoprotein.. Exp Cell Res.

[35] Sahai E, Marshall CJ (2003). Differing modes of tumour cell invasion have distinct requirements for Rho/ROCK signalling and extracellular proteolysis.. Nat Cell Biol.

[36] Mills JC, Stone NL, Erhardt J, Pittman RN (1998). Apoptotic membrane blebbing is regulated by myosin light chain phosphorylation.. J Cell Biol.

[37] Sebbagh M, Renvoize C, Hamelin J, Riche N, Bertoglio J (2001). Caspase-3-mediated cleavage of ROCK I induces MLC phosphorylation and apoptotic membrane blebbing.. Nat Cell Biol.

[38] Charras GT, Yarrow JC, Horton MA, Mahadevan L, Mitchison TJ (2005). Non-equilibration of hydrostatic pressure in blebbing cells.. Nature.

[39] Coleman ML, Sahai EA, Yeo M, Bosch M, Dewar A (2001). Membrane blebbing during apoptosis results from caspase-mediated activation of ROCK I.. Nat Cell Biol.

[40] Yoshida K, Soldati T (2006). Dissection of amoeboid movement into two mechanically distinct modes.. J Cell Sci.

[41] Barros LF, Kanaseki T, Sabirov R, Morishima S, Castro J (2003). Apoptotic and necrotic blebs in epithelial cells display similar neck diameters but different kinase dependency.. Cell Death Differ.

[42] Totsukawa G, Wu Y, Sasaki Y, Hartshorne DJ, Yamakita Y (2004). Distinct roles of MLCK and ROCK in the regulation of membrane protrusions and focal adhesion dynamics during cell migration of fibroblasts.. J Cell Biol.

[43] du Roure O, Saez A, Buguin A, Austin RH, Chavrier P (2005). Force mapping in epithelial cell migration.. Proc Natl Acad Sci U S A.

[44] Bear JE, Svitkina TM, Krause M, Schafer DA, Loureiro JJ (2002). Antagonism between Ena/VASP proteins and actin filament capping regulates fibroblast motility.. Cell.

[45] Mejillano MR, Kojima S, Applewhite DA, Gertler FB, Svitkina TM (2004). Lamellipodial versus filopodial mode of the actin nanomachinery: pivotal role of the filament barbed end.. Cell.

[46] Rottner K, Behrendt B, Small JV, Wehland J (1999). VASP dynamics during lamellipodia protrusion.. Nat Cell Biol.

[47] Galbraith CG, Yamada KM, Sheetz MP (2002). The relationship between force and focal complex development.. J Cell Biol.

[48] Sims TN, Soos TJ, Xenias H, Dubin-Thaler B, Hofman J (2007). Opposing effects of PKCtheta and WASp on symmetry breaking and relocation of the immunological synapse.. Cell.

[49] Fujinami N, Kageyama T (1975). Circus movement in dissociated embryonic cells of a teleost, Oryzias latipes.. J Cell Sci.

[50] Kageyama T (1977). MOTILITY AND LOCOMOTION OF EMBRYONIC CELLS OF THE MEDAKA, ORYZIAS LATIPES, DURING EARLY DEVELOPMENT.. Development, Growth & Differentiation.

[51] Keller HU, Bebie H (1996). Protrusive activity quantitatively determines the rate and direction of cell locomotion.. Cell Motil Cytoskeleton.

[52] Theriot JA, Mitchison TJ (1991). Actin microfilament dynamics in locomoting cells.. Nature.

[53] Bailly M, Condeelis JS, Segall JE (1998). Chemoattractant-induced lamellipod extension.. Microsc Res Tech.

[54] Chamaraux F, Fache S, Bruckert F, Fourcade B (2005). Kinetics of cell spreading.. Phys Rev Lett.

[55] Sawada Y, Tamada M, Dubin-Thaler BJ, Cherniavskaya O, Sakai R (2006). Force sensing by mechanical extension of the Src family kinase substrate p130Cas.. Cell.

[56] Vial E, Sahai E, Marshall CJ (2003). ERK-MAPK signaling coordinately regulates activity of Rac1 and RhoA for tumor cell motility.. Cancer Cell.

[57] Munevar S, Wang YL, Dembo M (2004). Regulation of mechanical interactions between fibroblasts and the substratum by stretch-activated Ca2+ entry.. J Cell Sci.

[58] Ayala R, Shu T, Tsai LH (2007). Trekking across the brain: the journey of neuronal migration.. Cell.

[59] Marin O, Valdeolmillos M, Moya F (2006). Neurons in motion: same principles for different shapes?. Trends Neurosci.

[60] Friedl P, den Boer AT, Gunzer M (2005). Tuning immune responses: diversity and adaptation of the immunological synapse.. Nat Rev Immunol.

[61] Wang W, Wyckoff JB, Goswami S, Wang Y, Sidani M (2007). Coordinated regulation of pathways for enhanced cell motility and chemotaxis is conserved in rat and mouse mammary tumors.. Cancer Res.

[62] Lacayo CI, Pincus Z, VanDuijn MM, Wilson CA, Fletcher DA (2007). Emergence of large-scale cell morphology and movement from local actin filament growth dynamics.. PLoS Biol.

[63] Zhang X, Jiang G, Cai Y, Monkley SJ, Critchley DR (2008). Talin depletion reveals independence of initial cell spreading from integrin activation and traction.. Nat Cell Biol.

[64] Shlomovitz R, Gov NS (2007). Membrane waves driven by actin and Myosin.. Phys Rev Lett.

[65] Church GM (2005). From systems biology to synthetic biology.. Mol Syst Biol.

[66] Hastie T (2001). The elements of statistical learning : data mining, inference, and prediction.

[67] Weiss Y, Fleet DJ (2000). Velocity likelihoods from generative models.. Investigative Ophthalmology&Visual Science.

[68] Poggio T, Torre V, Koch C (1985). Computational Vision and Regularization Theory.. Nature.

[69] Cai Y, Biais N, Giannone G, Tanase M, Jiang G (2006). Nonmuscle myosin IIA-dependent force inhibits cell spreading and drives F-actin flow.. Biophys J.

