# Supplementary material for: Quantification of Cell Edge Velocities and Traction Forces Reveals Distinct Motility Modules during Cell Spreading
Source: PLoS One. 2008 Nov 17;3(11):e3735. doi: 10.1371/journal.pone.0003735 (PMC2581916; doi:10.1371/journal.pone.0003735)
Supplement: Appendix S1 — From an analysis of membrane edge velocities, we estimate the kinetic constants of actin polymerization as a function of Cytochalasin D. (0.05 MB DOC) [file pone.0003735.s001.doc]

## Appendix S1

From an analysis of membrane edge velocities, we can estimate the kinetic constants of actin polymerization as a function of additives. Neglecting geometrical and other factors of order one, we get for the number of added monomers per unit time

,

where *V*_P1_ = 3.2 μm/min is the typical edge velocity in the P1 phase, and *l*_m_  = 2 nm is the length of filament extension from one actin monomer. With the known bare rate constant k_on_=10 /(μM s), and assuming

,

this corresponds to c_0_= 26/s x 0.1 μM s = 2.6 μM for the actin monomer concentration at the leading edge.

With a linear dependence of the overall actin polymerization rate dN/dt on CD concentration *c_CD_*

we find the two constants *k_0_*= 26/s and *k_1_* = - 0.16 / (nM s) from a fit to the mean velocity *μ_2_* versus CD concentration for P1. Note that it is important to disentangle the various contributions to the membrane velocity in order to obtain sensible rate constants. The rate constant k_1_ should be considered an effective quantity, since we didn´t measure cellular CD concentrations. Rather we used the concentration of externally added CD, which is likely to be larger than the intracellular concentration.
