# Supplementary figures and images for: Quantification of Cell Edge Velocities and Traction Forces Reveals Distinct Motility Modules during Cell Spreading
Source: PLoS One. 2008 Nov 17;3(11):e3735. doi: 10.1371/journal.pone.0003735 (PMC2581916; doi:10.1371/journal.pone.0003735)

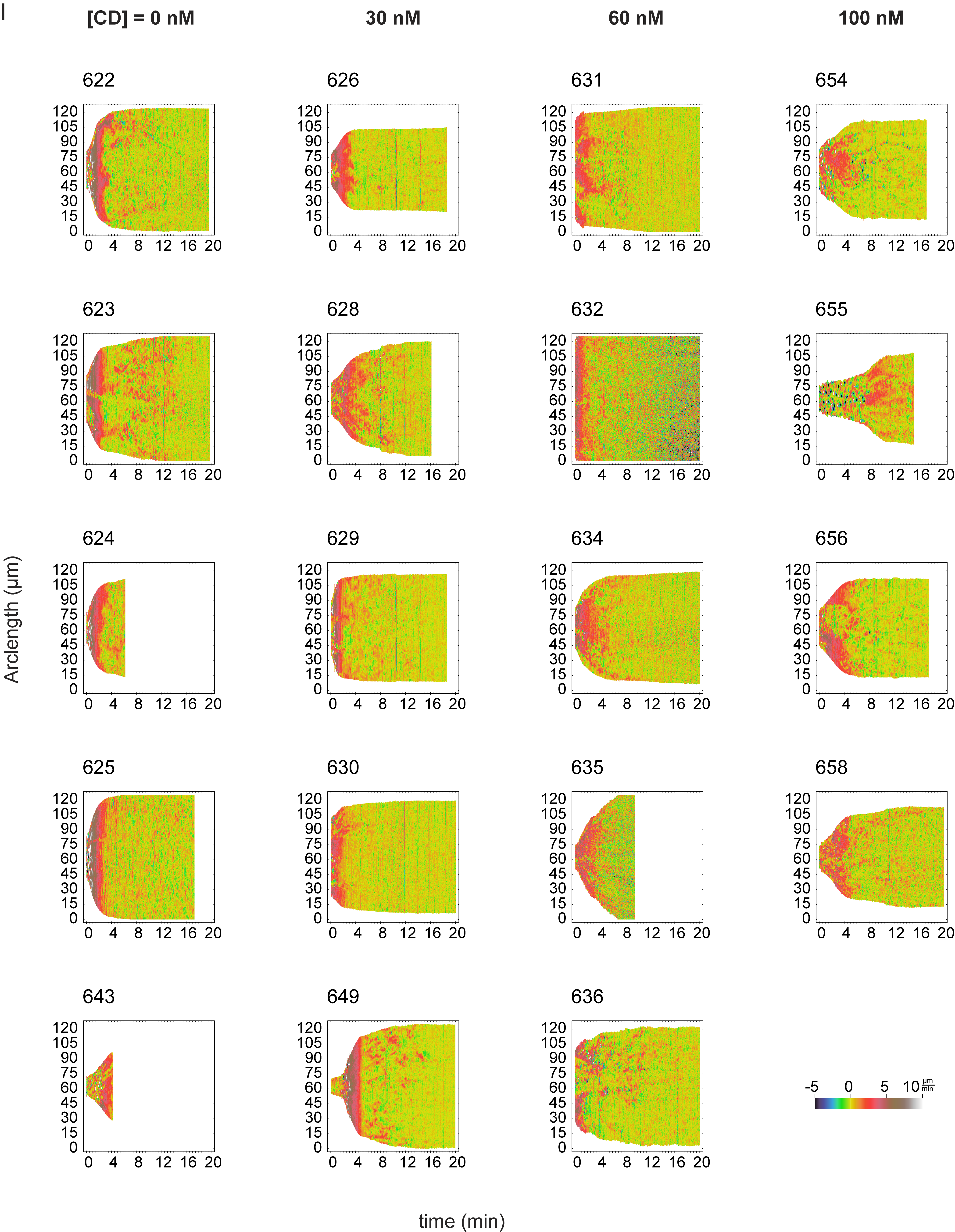

Supplement: Dataset S1 — Velocity vs. [CD] Part I. Part I of II. Velocity maps of isotropic cells used in the CD spreading dependence studies. The numbers above each plot indicates the cell ID # in our database. The data for these velocity plots, area vs. time curves, and sample algorithms for visualizing data are all accessible through http://cellmap.cellmotion.org/. (11.80 MB TIF) [file pone.0003735.s002.tif]

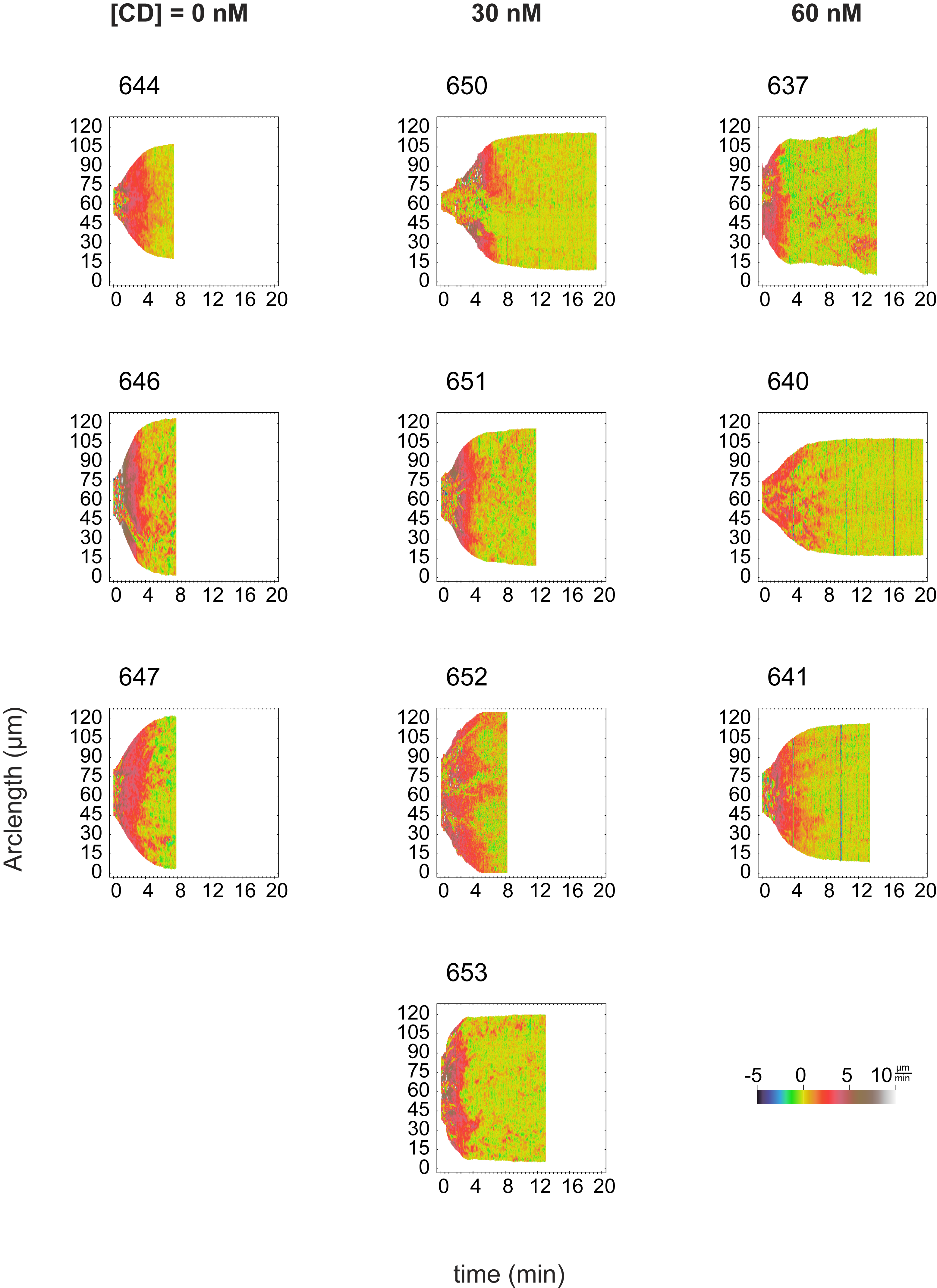

Supplement: Dataset S2 — Velocity vs. [CD] Part II. Part II of II. Velocity maps of isotropic cells used in the CD spreading dependence studies. The numbers above each plot indicates the cell ID # in our database. The data for these velocity plots, area vs. time curves, and sample algorithms for visualizing data are all accessible through http://cellmap.cellmotion.org/. (4.99 MB TIF) [file pone.0003735.s003.tif]
